# Field Performance and Calibration Strategies for Low-Cost Capacitive Soil Moisture Sensors

**DOI:** 10.3390/s26113291

**Published:** 2026-05-22

**Authors:** Glenn Strypsteen, Magnus Persson, Mykola Miroshnychenko, Nikola Rakonjac

**Affiliations:** 1 Division of Water Resources Engineering, Lund University, 221 00 Lund, Sweden; glenn.strypsteen@tvrl.lth.se; 2 The National Scientific Centre “Institute for Soil Science and Agrochemistry Research Named After O.N. Sokolovsky”, 61024 Kharkiv, Ukraine; ecosoil54@gmail.com; 3 Soil Physics and Land Management Group, Wageningen University, 6708 PB Wageningen, The Netherlands; nikola.rakonjac@wur.nl

**Keywords:** soil moisture, low-cost capacitance sensor, calibration, field experiments, irrigation management

## Abstract

**Highlights:**

**What are the main findings?**

- Low-cost capacitive soil moisture sensors can achieve high accuracy when properly calibrated, rivaling more advanced sensors under both laboratory and field conditions.
- A simple one-point calibration method is suggested, eliminating the need for labor-intensive multi-point field calibration.

**What are the implications of the main findings?**

- Cost-effective soil moisture monitoring becomes feasible at farm scale, enabling wider adoption of precision irrigation and data-driven water management.
- The proposed 1PC method provides a scalable calibration framework, enabling reliable low-cost sensor networks for precision agriculture and IoT-based soil monitoring.

**Abstract:**

Low-cost capacitive (LCC) soil moisture sensors have considerable potential for precision agriculture due to their low cost, low energy consumption, and suitability for IoT-based systems. However, their field performance and the transferability of laboratory calibration to field conditions remain insufficiently documented. The objective of this study was to evaluate the field performance of LCC sensors calibrated in the laboratory and to assess practical calibration strategies for field application. Laboratory calibration was performed for eight soil types, and field performance was evaluated in four experiments under different soil and climatic conditions. The sensors showed high accuracy under laboratory conditions, with RMSE values of 0.002–0.022 m^3^/m^3^ for soil-specific calibration, but substantially larger errors when laboratory-derived calibrations were applied directly in the field (RMSE 0.055–0.191 m^3^/m^3^). Soil-specific field calibration gave the highest accuracy (RMSE 0.005–0.036 m^3^/m^3^), whereas a simplified one-point calibration also improved performance considerably (RMSE 0.006–0.078 m^3^/m^3^) while requiring much less effort. Sensor performance declined at high water contents and under saline conditions. The results show that low-cost capacitive sensors can be used for reliable field soil moisture monitoring.

## 1. Introduction

Soil water content is a critical parameter in agriculture and environmental management, directly impacting plant growth, irrigation needs, and ecological processes. Moisture of porous materials is also a key variable in many other applications such as hydrology, geotechnical engineering, food engineering and building materials. Thus, accurate measurements of water content are very important for a wide range of applications.

Many techniques for water content measurements have been developed covering many different scales. Traditional techniques for water content measurements are, however, either labour intensive (e.g., gravimetric sampling) or require advanced and expensive equipment, e.g., time domain reflectometry (TDR) or frequency domain reflectometry (FDR). Until recently, systems of automated measurement equipment have been restricted to researchers or other users with a lot of resources. The limitations of traditional techniques have led to the development of low-cost sensors [1]. During the last decades, several capacitive and resistive sensors have been developed. These offer lower cost and consume less energy compared to, e.g., TDR or FDR sensors, but the trade-off is that they have lower accuracy and precision [2,3]. These low-cost sensors can be controlled by microcontrollers and have shown great potential for use in smart systems or Internet of Things (IoT) applications [4]. Some examples of such applications have been discussed by Bhavsar et al. [5], who presented a comprehensive review of smart drip and sprinkler irrigation systems. A system with low-cost capacitance sensors controlling RGB LED lights to indicate when irrigation was needed was designed by Pitoro et al. [6]. The current frontier lies in integrating capacitive sensors into IoT platforms for real-time data collection and decision support.

Most applications of low-cost capacitance sensors have been used in controlled environments in the laboratory or greenhouse. There are relatively few examples of published studies of field applications. Some examples of field applications can be found in Schwamback et al. [3], who measured soil water content under experimental plots of different land use in Itirapina, Brazil, and Adla et al. [7] who measured soil water content in an experimental wheat farm in Kanpur, India. Due to the limited number of field studies, there is a general lack of data from field applications needed for assessing the accuracy and precision of low-cost capacitance sensors in farm-scale applications.

When sensors are installed in the field, the common procedure is to first calibrate the sensors in the laboratory using repacked or undisturbed soil samples that are wetted or dried to known water contents. This laboratory calibration is then used in the field to convert sensor readings to water content. Rudnick et al. [8] tested three water content capacitance-based measurement techniques in a field study and compared performance to water content measurements using a neutron gauge. They concluded that field calibrations were better than the manufacturer’s laboratory-based calibration for all sensors. Singh et al. [9] evaluated in a field study eight water content sensors in reference to a neutron moisture meter. They concluded that calibrating against reference measurements, performance improved substantially beyond laboratory-based factory calibrations, with root mean square errors ranging from 0.039 to 0.157 m^3^/m^3^ for factory calibration to below 0.025 m^3^/m^3^ for all sensors using calibration against the reference method. Adla et al. [7] used low-cost capacitance sensors to improve the AquaCrop model performance. They concluded that calibrating the sensor in field conditions against a secondary standard sensor gave lower errors compared to laboratory calibration.

As presented by the studies mentioned above, calibrating low-cost sensors to a reference method improves field performance compared to laboratory calibration. On the other hand, calibrating against a reference method is labour intensive and, of course, requires a reference method to compare with. Thus, the lack of a simple and effective way of calibrating low-cost capacitance sensors for field use is limiting the use of these sensors in farm-scale environments.

The objective of this study was to evaluate the field performance of low-cost capacitive (LCC) sensors when calibrated in the laboratory and to assess practical calibration strategies suitable for field application. To this end, laboratory calibrations were conducted on eight different soil types. Two laboratory calibration methods and two field calibration methods were evaluated in four field experiments in different soil types and environments.

## 2. Materials and Methods

### 2.1. Hardware

**Figure 1:**
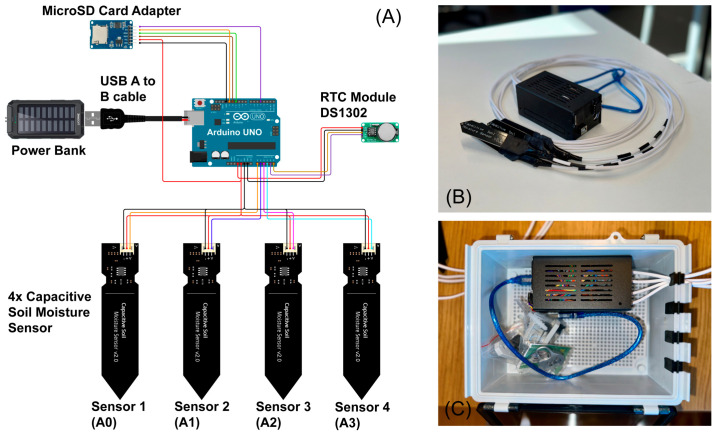
(**A**) Low-cost capacitance (LCC) sensor v2.0. (**B**) Soil moisture sensor outside the waterproof box, with the SD card slot positioned on the exterior of the Arduino casing. The four soil moisture probes, connected to the Arduino, are marked with black tape to indicate probes 1 through 4. (**C**) Soil moisture sensor inside the waterproof box, with the probe cables extending out of the enclosure. The power bank is also located within this box.

There are many different types of low-cost capacitance sensors on the market. There seem to be many similar sensors sold by many manufacturers for prices lower than €1 or $1, even cheaper in bulk. Documentation such as user manuals or technical specification is generally sparse or non-existent [4,10]. In the present study the Capacitive Soil Moisture Sensor (v2.0) was used, hereafter referred to as low-cost capacitive (LCC) sensor. A soil moisture monitoring system was built using four LCC sensors, controlled by an Arduino UNO microcontroller (Figure 1A). The system was powered by a power bank, and the output data was stored on an SD card. The system was similar to the one described by [3]; however, an updated version of the sensors was used in the present study. The sensors were waterproofed by hot glue and heat shrink tube to protect the otherwise exposed electronics at the top of the sensor. The waterproofing was tested by immersing the entire sensor in water for 24 h.

The Arduino UNO and electronics were enclosed in a metal case for protection (Figure 1B). The SD card slot is positioned on the exterior of the Arduino casing and the four probes connected to the Arduino are marked with black tape to indicate probes 1–4. Figure 1C shows the sensor inside the waterproof box with the probe cables extending out of the enclosure. The power bank is also housed within this enclosure.

### 2.2. How It Works

The LCC sensor uses a digital signal with a variable frequency between 260 and 520 Hz depending on the capacitance of the surrounding medium (soil) in response to changes in volumetric water content (VWC), which is filtered through an RC circuit to provide an analogue DC output value. See [4,10] for a thorough theoretical description of the sensor.

### 2.3. Laboratory Calibration

Laboratory calibration was performed in oven-dried sieved soil (<2 mm) using eight different soil types. The soil types used are listed in Table 1.

The samples were packed into cylinders with a volume of 600 cm^3^ to its field bulk density. Each soil sample was wetted incrementally with an increment of 0.042 m^3^/m^3^, to predefined moisture contents, ranging from oven dry to near saturation, 0.33 m^3^/m^3^. All soil types were calibrated using the same laboratory method, except for the silty clay loam, which was prepared in a different laboratory and for which only VWCs of 0, 0.10, 0.15, and 0.20 m^3^/m^3^ were used, with 0.20 m^3^/m^3^ being the maximum water content. In each sample, five analogue sensor output (SO) values were measured using each of four sensors. The average and standard deviation of the 20 measurements at each water content were recorded. In the sandy clay loam and the loamy sand measurements were also taken with a WET-sensor and a ThetaProbe probe ML3 (both from Delta-T Devices Ltd., Cambridge, United Kingdom). The bulk electrical conductivity was measured in the soils collected in field using the WET-sensor. All soils could be classified as non-saline. The influence of soil salinity was investigated in the clay loam soil by repeating the calibration with saline solutions with a NaCl concentration of 3, 5, and 10 g/L.

### 2.4. Field Tests

Four field tests were conducted. The details about the sites can be found in Table 2. Two field experiments were carried out in Gårdstånga Nygård, southern Sweden. The first (GN1) during an uncropped period and the second (GN2) in an irrigated rapeseed (*Brassica napus* L.) cultivation field. Experiment GN1 was conducted in a soil column, 0.25 m in diameter, with repacked soil taken from the field. Four sensors were installed, two at 0.1 m depth (sensor 2 and 4) and two at 0.3 m depth (sensor 1 and 3). The two sensors at each depth were placed approximately 0.1 m apart. A WET-sensor was installed at the 0.1 m depth, approximately 0.1 m beside one of the LCC sensors. The soil column was buried with the soil surface at the same level as the surrounding soil. The column was exposed to the atmosphere at the top while the bottom was in contact with the soil below. During this experiment, six measurements were taken and averaged once every hour. WET-sensor measurements were taken weekly when the power banks were changed, but also at irregular intervals in between. In total 68 WET-sensor measurements were taken.

**Figure 2:**
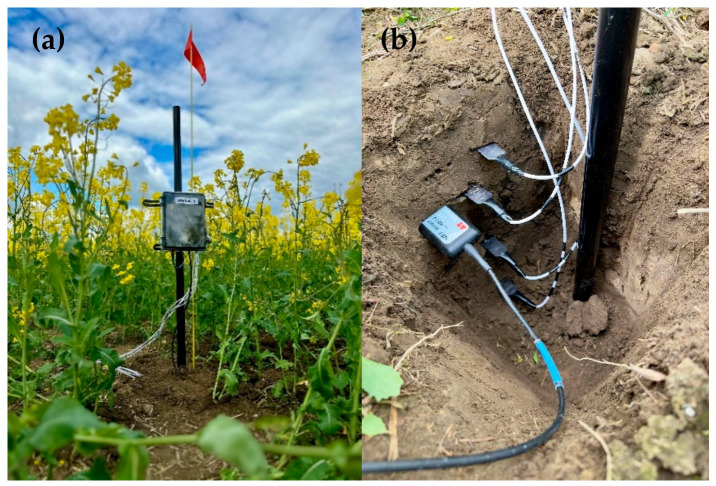
Field installation of the soil moisture monitoring system during experiment GN2. (**a**) Monitoring unit installed in a rapeseed field, including enclosure, mounting pole, and sensor cables. (**b**) Subsurface installation showing the low-cost capacitive (LCC) sensors placed at multiple depths and a co-located WET sensor used as reference.

In experiment GN2, two systems were installed in the field (GN2.1 and GN2.2) 5 m apart, with LCC sensors placed at depths of 0.05, 0.10, 0.20, and 0.30 m (see Figure 2). Two ThetaProbe probes, one at each system, were buried at the 0.10 m depth, close to the LCC sensors at that depth. The sensors were measured every minute, but the measurements were later aggregated to hourly averages for analysis. The manual ThetaProbe measurements were collected weekly when the power banks were replaced.

In the field site in Ukraine (UKR, Table 2), two field experiments were carried out, one in 2024 and one in 2025. One system with four sensors were used. The sensors were installed at two depths, 0.1 and 0.9 m, respectively, in two profiles with one sensor at each depth. The two profiles were located around 2 m apart. Due to unstable power supply in the field, the sensors were actively measuring only when there were staff in the field. Reference water content measurements were taken using the gravimetric method by drying soil samples at 105 °C. For this, a soil drill was used to collect soil samples at two depths at each profile. During 2024, there was a prolonged drought leading to that no crops were grown at the field site. Thus, soil sampling was only performed twice. During 2025 soil samples were taken six times between 10 March and 3 June. In total there were 32 pairs of sensor readings and water contents. In the following, the data from both periods were aggregated to produce a larger data set, resulting in 10 gravimetric samples taken per sensor.

One field experiment was carried out during the period 21 May to 18 June 2025 in Torna Hällestad (TH, Table 2), Sweden, in a field where garlic (*Allium sativum*, L.) was cultivated. One system with four sensors was used. The sensors were installed in two rows, one in line with the plant rows and one in between two plant rows. In each row, one sensor was installed at 0.10 and 0.30 m depth, respectively. Close to the sensor at 0.10 m depth between the plant rows, a WET-sensor was buried. With this sensor, reference water content measurements were taken manually once or twice a day, resulting in 40 measurements.

### 2.5. Calibration Methods

In the present study, four different calibration methods were tested and evaluated. The first is the soil specific calibration based on laboratory data, hereafter abbreviated SSCL. This method is based on the sensor measurements taken in each soil type in the laboratory. Different types of calibration equations have been suggested in the literature, linear [3,11,12], polynomial [4,12,13,14], machine learning [15], and exponential [12,16]. In the present study, we tested a linear, two- and three-order polynomial and exponential calibration equations. The exponential calibration equation was found to give the best results and consequently, the following calibration equation was used:

(1) $$SO=a+b\times e^{\left(-VWC/c\right)}$$

(2) $$\mathrm{VWC}=-\ln\left(\frac{SO-a}{b}\right)\times c$$

where SO is the analogue sensor output, VWC is the water content in m^3^/m^3^, and a, b, and c are empirical parameters. In the SSCL method, the best fit parameters of Equation (2) were calculated for each soil type individually.

The second calibration method was the universal calibration based on laboratory data, hereafter abbreviated UCL. In this method, data from all soil types were combined into a single dataset and the best fit parameters of Equation (2) were calculated.

The third calibration method was the soil specific calibration using field data, hereafter abbreviated SSCF. In this method, the VWC measured by the reference method obtained in the field was related to the SO from the sensor(s) installed close to the location of the reference measurement and the best fit parameters of Equation (2) were calculated for each sensor/reference measurement combination.

To simplify the calibration of field sensors, a one-point calibration method is proposed, hereafter abbreviated 1PC. This method is based on taking one water content measurement measured with the reference method in the field close to where the LCC sensor is installed. All other LCC sensor readings are calculated using the water content difference compared to the reference measurement calculated using the universal calibration curve determined in the laboratory. The idea behind the one-point calibration is that using the universal calibration the relative changes can be estimated. In this method the water content is calculated by

VWC_1PC_ = VWC_UCL_ + (VWC_ref_ − VWC_ref,UCL_) (3)

where VWC_1PC_ is the water content calculated using the 1PC, VWC_ref_ is the water content measured with the reference method, VWC_ref,UCL_ is the water content calculated using the UCL and the sensor output when the VWC_ref_ was taken and VWC_UCL_ is the water content calculated for any time.

## 3. Results

### 3.1. Laboratory Calibration

**Figure 3:**
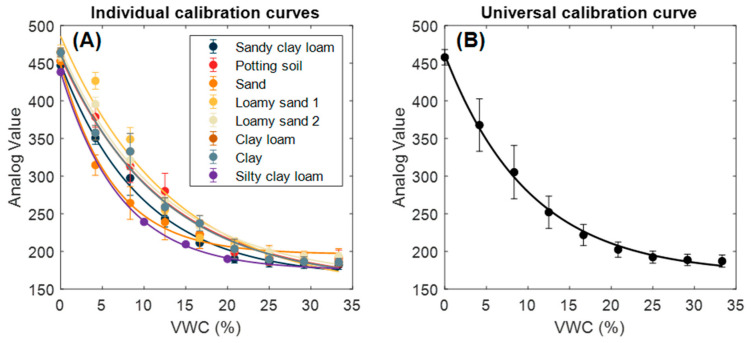
(**A**) Individual calibration curves relating analogue sensor output to volumetric water content (VWC) for eight soil types. Points represent mean analogue values at each imposed VWC, and error bars indicate ±1 standard deviation across replicate measurements. Solid lines show exponential fits for each soil. (**B**) Combined calibration curve obtained by combining data from all soils. Points represent the mean analogue response (±1 standard deviation), and the solid line shows the fitted exponential relationship used for the universal calibration. Across soil types, analogue output decreases nonlinearly with increasing VWC, with responses converging toward a lower detection limit at higher VWCs.

Figure 3A shows the individual calibration curves for the different soils. Each data point represents the mean analogue output value of five replicate measurements of four individual sensors. The error bars indicate the standard deviation of these measurements. The analogue output decreases nonlinearly with increasing VWC, and the response for all soils follows a similar exponential trend despite minor differences in slope and curvature between soil types. The high degree of similarity between soils justifies combining the data into a single universal calibration curve (Figure 3B), which forms the basis for the UCL approach.

During calibration, we found a clear detection limit of the sensors. In dry soils, analogue sensor output values typically approached 450–480 for dry soil, while for wet conditions, the probes did not register values below approximately 182–190 depending on soil type. This lower threshold indicates that the sensors are unable to fully resolve additional increases in soil VWC once this limit is reached. As a result, sensor sensitivity decreases at higher VWCs (i.e., above ~25%), which constrains the reliable measurement range of the probes in wetter soils.

The coefficient of determination (R^2^) and root mean square error (RMSE) of the calibration methods employed for the laboratory data, i.e., SSCL and UCL, are presented in Table 3 along with the best fit parameters of the SSCL (Equation (2)). The performance of the LCC sensor in terms of R^2^ and RMSE was similar to previously published results for similar sensors, although direct comparison of RMSE values should be done with caution due to different experimental procedures and range in VWC. The range in published RMSE is rather large, 0.009 to 0.078 m^3^/m^3^ [3,11,13,14,17], but many of these studies report RMSEs of <0.04 m^3^/m^3^.

.

With all measurements combined, a universal calibration curve was derived with best-fit parameters a = 170.7, b = 296.8, and c = 0.1. The UCL method resulted in an RMSE of 0.028 m^3^/m^3^ and an R^2^ of 0.93, indicating good overall agreement across soils.

The precision of the sensors was evaluated by calculating the standard deviation of the VWC estimation. The standard deviation was determined both for the five replicate measurements at each VWC for each of the four sensors in every soil type, and for all measurements from the four sensors combined. For each soil type, the average standard deviation was calculated as the mean of the standard deviations obtained at each VWC, for both individual sensor data and the pooled dataset. The results are presented in Table 4. Overall, the findings indicate low sensor-to-sensor variability, and in the present study there was no need for individual calibration of each sensor. The higher standard deviation for the pooled data reflects variability introduced when measurements from several sensors and water contents are combined and should therefore not be interpreted solely as sensor-to-sensor variability. In addition, small local differences in soil packing and soil conditions around each sensor may also have contributed to the pooled variability. Comparable standard deviation values for combined data were reported by Chereches et al. [18], who evaluated 28 sensors of the same type used in this study.

**Figure 4:**
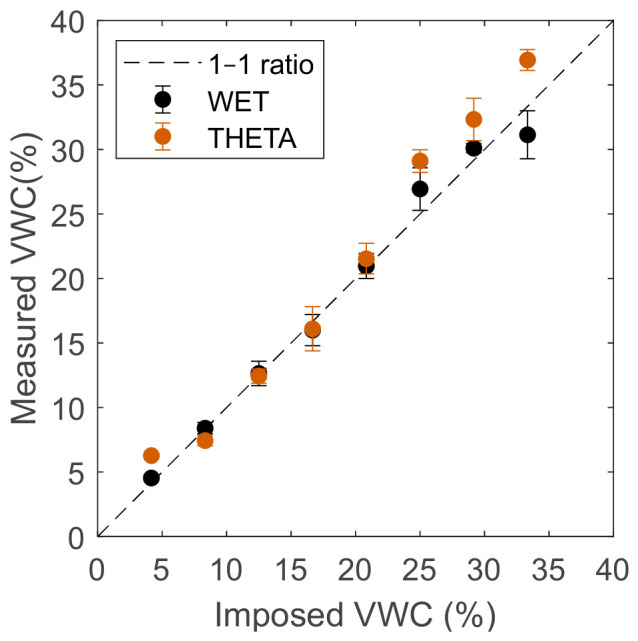
Comparison between imposed volumetric water content (VWC) and measurements obtained with the WET sensor (black circles) and ThetaProbe (orange circles) in sandy clay loam. The solid line represents the 1:1 relationship. Error bars indicate ±1 standard deviation of replicate measurements. Both sensors closely follow the 1:1 line across the tested moisture range, demonstrating good agreement with the imposed VWCs.

The WET sensor and the ThetaProbe provided accurate VWC estimations when using the default manufacturer calibration settings. In the sandy clay loam soil, the WET sensor showed excellent agreement with the imposed VWCs (R^2^ = 0.99, RMSE = 0.011 m^3^/m^3^), while the ThetaProbe also performed well (R^2^ = 0.98, RMSE = 0.024 m^3^/m^3^). As shown in Figure 4, both reference sensors closely followed the 1:1 line across the tested moisture range, indicating minimal systematic bias. The error bars represent ±1 standard deviation of the replicate measurements, as described previously for the laboratory calibration experiments.

Given this strong agreement, no additional soil-specific calibration was applied to the WET sensor or the ThetaProbe in this study. The performance of the ThetaProbe is consistent with previous findings reported by [19], who observed RMSE values in the range 0.011–0.054 m^3^/m^3^ using manufacturer calibration, and [20], who reported RMSE values below 0.0523 m^3^/m^3^. Similarly, the WET sensor performance is in line with results reported by [21,22].

### 3.2. Salinity Effects

**Figure 5:**
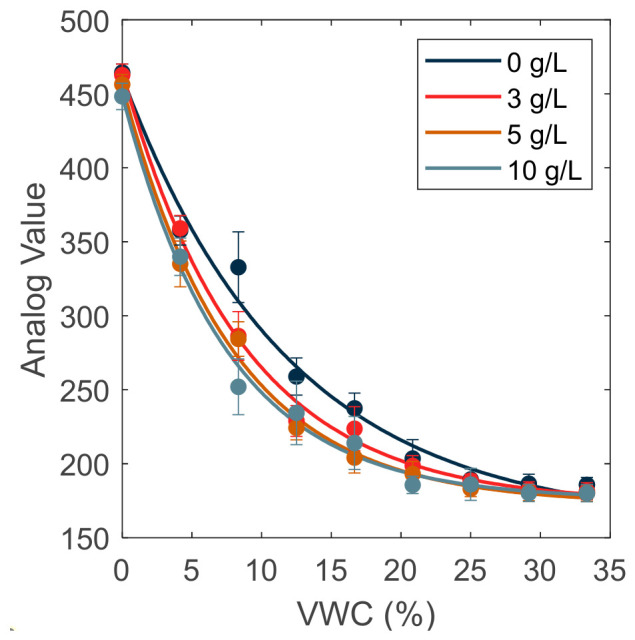
Effect of salinity on the analogue output–volumetric water content (VWC) relationship for the low-cost capacitive sensor. Calibration curves are shown for NaCl concentrations of 0, 3, 5, and 10 g/L. Points represent mean analogue values at each imposed VWC level, and error bars indicate ±1 standard deviation of replicate measurements. Increasing salinity shifts the calibration curves downward, resulting in lower analogue outputs at a given VWC and indicating a systematic underestimation of VWC when using a non-saline calibration curve.

Figure 5 illustrates the influence of increasing salinity on the analogue sensor response. The general effect of salinity is a systematic decrease in analogue output with increasing NaCl concentration at a given VWC. As salinity increases from 0 to 10 g/L, the calibration curves shift downward, particularly at intermediate moisture levels. This indicates that saline conditions cause the sensor to underestimate VWC when using a calibration curve derived under non-saline conditions.

This behaviour is consistent with previous findings [23,24,25] and reflects the influence of increased electrical conductivity on the dielectric response measured by capacitive sensors. Peddinti et al. [24] found that salinity effects on a low-cost resistivity-capacitance sensor became less pronounced in finer-textured soils such as clay loam compared to sandy soils, underscoring the interplay between salinity and soil physical properties. Gómez-Astorga et al. [13] demonstrated that calibration curves developed under non-saline conditions can deviate by up to ~30% when salinity is present, particularly at moderate to high salinity levels. In our case (Figure 5), the deviation from the non-saline calibration curve reached approximately 15% at a NaCl concentration of 10 g/L, especially at intermediate VWCs.

To assess the practical implications of salinity, soils were classified according to the USDA salinity classification system [26], which categorizes soils based on the electrical conductivity of the saturation extract (EC_s_). The EC_s_ was calculated from the electrical conductivity of the soil water (EC_w_) using

EC_s_ = VWC × EC_w_/VWC_s_ (4)

where VWC_s_ is the volumetric water content at saturation. Based on the calculated EC_s_ values, each sample was assigned to a USDA salinity class. The analogue sensor outputs were converted to VWC using the universal calibration curve, and performance metrics (R^2^ and RMSE) were determined for each salinity category.

The results show that sensor performance deteriorates with increasing salinity. In non-saline soils (EC_s_ < 2 dS/m), the universal calibration performed well (R^2^ = 0.96, RMSE = 0.017 m^3^/m^3^). In slightly saline soils (2–4 dS/m), performance decreased substantially (R^2^ = 0.80, RMSE = 0.043 m^3^/m^3^). In moderately saline soils (4–8 dS/m), the RMSE increased further to 0.048 m^3^/m^3^, although R^2^ remained relatively high (0.94). No data were available for highly saline soils (>8 dS/m).

Overall, the experimental results demonstrate that increasing salinity introduces systematic bias and increased uncertainty in VWC estimation. Consequently, the use of the low-cost capacitive (LCC) sensor is recommended primarily for soils classified as non-saline according to the USDA classification, i.e., soils with ECs below 2 dS/m.

The salinity analysis in the present study was limited to NaCl solutions and therefore represents a simplified case compared to the more complex ionic composition of natural soil solutions under field conditions. In practice, soil salinity may reflect varying proportions of different dissolved ions, which can influence electrical conductivity and sensor response in different ways. The results should therefore be interpreted as an indication of the general sensitivity of the LCC sensor to salinity rather than as a complete description of its performance under all saline field conditions.

### 3.3. Field Tests

**Figure 6:**
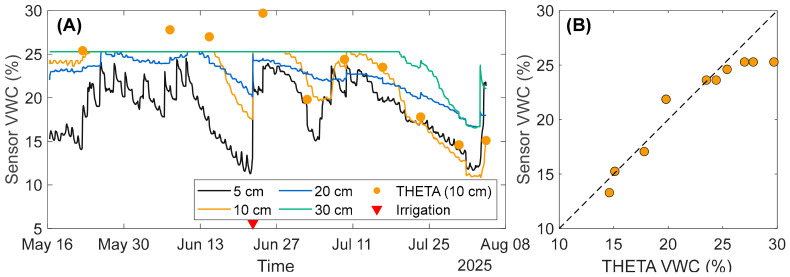
Field performance of the low-cost capacitive (LCC) sensor during experiment GN2.1. (**A**) Time series of volumetric water content (VWC) measured at 5, 10, 20, and 30 cm depths between mid-May and early August 2025. Orange markers represent weekly manual measurements with the ThetaProbe at 10 cm depth, and the red triangle indicates an irrigation event. (**B**) Relationship between LCC-derived VWC and ThetaProbe VWC at 10 cm depth; the dashed line represents the 1:1 line. Overall, the LCC sensor captures temporal moisture dynamics well, with deviations mainly occurring at higher VWCs.

All field tests produced useful time series of VWC. A quantitative analysis showed that the evolution of VWC over time was as expected, i.e., VWC increased after rainfall or irrigation and decreased during dry periods. As an example, the VWC over time for one of the systems (GN2.1) in experiment GN2 is shown in Figure 6A. The VWC values were calculated using the SSCF.

In GN2, the time series clearly capture distinct wetting events, followed by progressive drying phases. The shallow sensors (5 and 10 cm) respond rapidly to rainfall and irrigation, showing sharp increases and stronger short-term variability. In contrast, the deeper sensors (20 and 30 cm) exhibit a smoother and delayed response, reflecting vertical water redistribution and attenuation of surface forcing with depth. It should be noted that these sensors did not record volumetric VWC higher than ~25%, consistent with the upper detection limit observed in the laboratory calibration. The overall consistency between depths and the agreement with manual ThetaProbe reference measurements at 10 cm, shown in Figure 6B, confirms that the system reliably captures field-scale soil moisture dynamics.

In each field test, VWC was calculated using the four calibration approaches: soil-specific calibration in the laboratory (SSCL), universal calibration in the laboratory (UCL), soil-specific calibration in the field (SSCF), and one-point calibration (1PC). Table 5 summarizes the performance of all calibration methods across the experiments in terms of RMSE.

The 1PC can be performed using any of the measured reference VWCs (VWC_ref_) obtained for a given experiment and sensor. Consequently, the number of possible 1PC realizations corresponds to the number of available VWC_ref_ measurements. For each field test, the minimum, mean, and maximum RMSE obtained from all possible 1PC combinations are reported.

Across the experiments, SSCF consistently provided the best performance, yielding RMSE values below 0.021 m^3^/m^3^ in 8 out of 9 field tests. The second-best method was the mean 1PC, which ranked second in 8 out of 9 experiments. Notably, in 7 out of 9 field tests, even the worst-performing 1PC resulted in lower RMSE than both SSCL and UCL. The highest RMSE values were generally observed for SSCL, with one exception.

To assess the robustness of the 1PC approach, a trend analysis was conducted to evaluate whether RMSE depended on time or VWC. A significant trend (α = 0.05) was detected in only two experiments. In GN1, RMSE increased slightly over time, which may be attributed to gradual structural changes in the repacked soil. In TH, RMSE increased with higher VWCs, possibly due to the relatively wide VWC range and the reduced sensitivity of the LCC sensor at high VWC levels.

In all field tests except GN2, SSCL and UCL generally underestimated VWC. The discrepancies between laboratory- and field-derived calibrations likely arise from several factors, with differences in bulk density being a major contributor, as bulk density has been shown to significantly influence the calibration of low-cost capacitive sensors [4]. However, no bulk density measurements were made directly around the sensor locations during the field experiments to verify this assumption. It should also be noted that in the laboratory experiments, LCC-derived VWC was compared to gravimetrically determined VWC, whereas in the field experiments it was compared to values obtained from reference sensors. However, separate laboratory calibrations of the WET sensor and the ThetaProbe demonstrated that these reference methods provided accurate VWC measurements for the soils used in the field tests.

## 4. Discussion

The objectives of this study were (i) to evaluate the performance of laboratory calibration methods for low-cost capacitive (LCC) soil moisture sensors under field conditions and (ii) to develop and assess a practical field calibration method that improves measurement accuracy while remaining feasible for routine application.

The LCC water content sensor can produce high-quality measurements with low errors under the right conditions. In the laboratory calibration experiment, the RMSE ranged from 0.013 to 0.022 m^3^/m^3^ for different soil types when using a soil-specific calibration (SSCL), and from 0.018 to 0.032 m^3^/m^3^ when a universal calibration curve (UCL) was employed.

The exponential calibration function provided the best fit to the observed sensor response and was therefore selected. Although this equation is empirical, previous studies have shown that similar sensors can also be interpreted using more physically based approaches, where sensor output is related to bulk dielectric permittivity and subsequently converted to volumetric water content using dielectric models (e.g., [14]). Such approaches provide a stronger physical basis and may improve generality. However, in the present study, the empirical approach was considered more suitable, since the main objective was to obtain a robust and practical calibration for field application.

During the laboratory experiment, a limitation in measuring high VWCs (>0.25 m^3^/m^3^) was observed, which restricts the usable measurement range. One common application of LCC sensors is irrigation control. Irrigation is typically applied when soil VWC falls below field capacity (FC) and should stop when FC is reached. The lowest reliable reading of the LCC sensor is around 185, which corresponds to approximately 0.25 to 0.30 m^3^/m^3^ using the universal calibration curve presented in this study. Therefore, the LCC sensor is generally recommended only for soils with a field capacity of around 0.30 m^3^/m^3^ or less, the exact value depends on soil type and bulk density. Based on the relationship developed by Saxton et al. [27], this corresponds to a clay content of approximately 35%. Consequently, for irrigation control, the sensor is best suited for soils with less than 35% clay. The limited measurement range of the LCC sensor is therefore an important practical constraint. Since the sensor response became unreliable above approximately 0.25–0.30 m^3^/m^3^, its applicability is restricted in soils where field capacity exceeds this range, particularly in finer-textured soils with higher clay content. In such cases, the sensor may still be useful for tracking relative drying trends, but it is less suitable for accurately determining when the soil has reached field capacity or for controlling irrigation close to wet conditions. The sensor is therefore most suitable for coarse- to medium-textured non-saline soils, where the relevant water content range falls within the reliable measurement interval.

The influence of salinity was tested under laboratory conditions. Increasing salinity resulted in larger measurement errors. Due to this sensitivity, the LCC sensor is recommended only for soils classified as non-saline according to the USDA classification, i.e., soils with an electrical conductivity of the saturation extract below 2 dS/m. It should be noted that the salinity analysis was limited to NaCl solutions and therefore represents a simplified case compared with the more complex ionic composition of natural soil solutions under field conditions.

To evaluate whether laboratory-derived calibrations can be reliably applied under field conditions, eight field tests were conducted under different environmental and climatic conditions. The field experiments demonstrated that while the LCC sensor can produce stable and consistent long-term measurements, laboratory-based calibrations (both SSCL and UCL) did not always maintain the same level of accuracy in the field. This confirms that laboratory calibration alone is insufficient to guarantee optimal field performance.

Four calibration methods were tested for the field data: soil-specific laboratory calibration (SSCL), universal laboratory calibration (UCL), soil-specific field calibration (SSCF), and one-point calibration (1PC) using field data. A notable result was that the universal laboratory calibration performed better than the soil-specific laboratory calibration in all field experiments. A likely explanation is that the field conditions did not correspond closely enough to the laboratory calibration conditions for the specific soil, particularly with respect to bulk density, soil structure, and sensor installation. In that case, the universal calibration may have performed better because it represents an average response across several soils and conditions and was therefore less sensitive to mismatches between laboratory and field conditions. The result suggests that transferability from laboratory to field may be more important than soil specificity alone. It should also be noted that the description of soil properties was limited, and that texture was the only parameter consistently reported for all soils. Other properties, such as bulk density, organic matter content, and porosity, may also influence sensor response. This should be recognized as a limitation of the study, since a more complete description of soil properties would have improved the interpretation and transferability of the results. The best overall accuracy was nevertheless obtained using soil-specific calibration based on field data (SSCF), indicating that soil-specific calibration can be highly effective when it is based on measurements made under the actual field conditions. However, this approach requires multiple reference measurements and is therefore more labour intensive than the other methods.

The comparison between calibration methods in the present study was primarily based on R^2^ and RMSE, since these metrics provide a direct and practically relevant assessment of calibration performance in terms of VWC. However, the results also indicated systematic deviations between methods, as SSCL and UCL generally underestimated VWC in most field experiments. A more extensive statistical evaluation could therefore have provided additional information on the nature of the calibration errors. However, this was beyond the scope of the present study.

Although the WET sensor and ThetaProbe were used as reference methods in the field experiments, they are not uncertainty-free, and their accuracy may vary with soil conditions and calibration. In the present study, both sensors showed good agreement with VWC in the laboratory, which supported their use as reference methods in the field soils investigated. It should nevertheless be acknowledged that different reference approaches were used in laboratory and field conditions, with gravimetric measurements serving as the laboratory standard and the WET sensor, ThetaProbe, or gravimetric sampling used in the field. This may have introduced some inconsistency between experiments, but the separate evaluation of the reference sensors indicated that their uncertainty was small relative to the differences observed between calibration methods.

The one-point calibration method, based on a single reference measurement combined with the universal laboratory calibration equation, provided very good accuracy while requiring minimal manual effort. If the reference method is gravimetric soil sampling, only a household scale and an oven are required, making the method accessible and practical for widespread use.

## 5. Conclusions

This study showed that low-cost capacitive (LCC) soil moisture sensors can provide accurate measurements under laboratory conditions but that laboratory calibration alone is not sufficient to ensure reliable field performance. Thus, the first objective was met by demonstrating that field errors increased substantially when laboratory-derived calibrations were applied directly under field conditions. The second objective was met by showing that field-specific calibration improves accuracy and that the proposed one-point calibration offers a practical compromise between accuracy and effort. The results also showed that the sensor is mainly suitable for non-saline soils and for water contents below approximately 0.25–0.30 m^3^/m^3^. Overall, the study demonstrates that low-cost capacitive sensors can be useful for field soil moisture monitoring, provided that their limitations are acknowledged and a simple field calibration is applied.

## Figures and Tables

**Table 1:** Soils used for laboratory calibration.

Texture	Bulk Density (g/cm^3^)	Origin
Sand	1.54	Fine sand, commercial source
Potting soil	1.18	Peat based potting soil, commercial source
Sandy clay loam	1.14	Gårdstånga, Sweden
Loamy sand 1	1.26	Torna Hällestad, Sweden
Loamy sand 2	1.27	Wageningen, The Netherlands
Clay loam	1.10	Lund, Sweden
Clay	1.03	Dalby, Sweden
Silty clay loam	1.18	Kharkiv, Ukraine

**Table 2:** A summary of the field tests of the LCC sensor.

Experiment	Period	Crop	# Sensors	Sensor Depths (m)	Reference Measurements
GN1	10 July 2024 to 19 October 2024	None	4	0.10; 0.30	WET sensor
GN2	16 May 2025 to 1 August 2025	Rapeseed	8	0.05; 0.10; 0.20; 0.30	THETA probe
TH	21 May 2025 to 18 June 2025	Garlic	4	0.10; 0.30	WET sensor
UKR	April 2024 to September 2024 10 March 2025 to 3 June 2025	None/Vegetables	4	0.10; 0.90	Gravimetric sampling

**Table 3:** Calibration performance of the low-cost capacitive (LCC) sensor across eight soil types. Results are shown for the universal laboratory calibration (UCL) and the soil-specific calibration based on laboratory data (SSCL). For each method, the coefficient of determination (R^2^) and root mean square error (RMSE, m^3^/m^3^) are reported. The coefficient of variation (CV, %) represents the relative variability of the estimated VWC. The fitted parameters (a, b, c) correspond to the exponential soil-specific calibration equation (Equation (2)). The universal calibration equation used is VWC=−ln((SO−170.7)/296.8)×0.1.

Soil Texture	UCL		SSCL		CV	SSCL Parameters		
	R^2^	RMSE	R^2^	RMSE		a	b	c
		m^3^/m^3^		m^3^/m^3^	%			
Sand	0.96	0.032	0.99	0.008	2.57	187.29	224.96	0.086
Potting soil	0.95	0.026	0.96	0.022	5.33	187.20	415.90	0.062
Sandy clay loam	0.97	0.031	0.99	0.013	3.66	180.20	364.30	0.062
Loamy sand 1	0.97	0.021	0.99	0.013	2.73	180.49	374.51	0.079
Loamy sand 2	0.97	0.030	0.96	0.021	2.25	164.40	304.60	0.123
Clay loam	0.97	0.018	0.97	0.017	4.10	175.52	332.08	0.088
Clay	0.98	0.032	0.98	0.015	2.61	178.00	288.40	0.071
Silty clay loam	0.99	0.050	0.99	0.002	1.27	171.60	264.59	0.076

Note: UCL = universal calibration based on laboratory data; SSCL = soil-specific calibration based on laboratory data; RMSE = root mean square error; CV = coefficient of variation; VWC = volumetric water content. The fitted parameters *a*, *b*, and *c* refer to Equation (2). The universal calibration equation used was VWC=−ln((SO−170.7)/296.8)×0.1.

**Table 4:** Standard deviation of estimated VWC (in m^3^/m^3^) using soil specific calibration based on laboratory data (SSCL) for individual sensors and pooled data across soil types.

Soil Texture	Sensor 1	Sensor 2	Sensor 3	Sensor 4	Pooled
Sand	0.0021	0.0091	0.0080	0.0028	0.0343
Potting soil	0.0013	0.0059	0.0008	0.0019	0.0253
Sandy clay loam	0.0019	0.0066	0.0014	0.0022	0.0188
Loamy sand 1	0.0016	0.0074	0.0014	0.0017	0.0262
Loamy sand 2	0.0013	0.0016	0.0020	0.0013	0.0125
Clay loam	0.0016	0.0031	0.0047	0.0018	0.0271
Clay	0.0022	0.0029	0.0027	0.0026	0.0142
Silty clay loam *	0.0007	-	-	-	-

Note: VWC = volumetric water content; SSCL = soil-specific calibration based on laboratory data. Values represent the standard deviation of estimated VWC for individual sensors and pooled data across soil types. * Only one sensor was used in this soil during the laboratory calibration experiment.

**Table 5:** Root mean square error (RMSE, m^3^/m^3^) of the different calibration methods in the field tests. For the one-point calibration (1PC), the minimum, mean, and maximum RMSE of all possible realizations are shown. n denotes the number of possible 1PC realizations. The VWC range indicates the observed VWC range during each field test.

Field Test	SSCL	UCL	SSCF	1PC Min	1PC Mean	1PC Max	n (1PC)	VWC_min_	VWC_max_
GN2.1	0.056	0.016	0.007	0.010	0.014	0.027	7	14.6	25.4
GN2.2	0.058	0.012	0.005	0.006	0.008	0.011	6	18.1	25.0
UKR 1–10	0.036	0.042	0.018	0.058	0.061	0.071	6	6.0	27.0
UKR 2–10	0.057	0.064	0.020	0.025	0.034	0.048	8	5.5	25.4
UKR 1–90	0.056	0.065	0.021	0.023	0.030	0.048	7	9.9	22.0
UKR 2–90	0.082	0.099	0.036	0.044	0.060	0.078	7	9.5	21.1
GN1	0.191	0.144	0.006	0.006	0.008	0.019	75	32.8	38.0
TH	0.136	0.135	0.006	0.022	0.029	0.057	40	23.4	38.2

Note: SSCL = soil-specific calibration based on laboratory data; UCL = universal calibration based on laboratory data; SSCF = soil-specific calibration based on field data; 1PC = one-point calibration; RMSE = root mean square error; VWC = volumetric water content. For 1PC, the minimum, mean, and maximum RMSE values of all possible realizations are shown. n denotes the number of possible 1PC realizations, and VWC_min_ and VWC_max_ indicate the observed VWC range during each field test.

## Data Availability

The original data presented in the study are openly available in https://zenodo.org/records/16911016 (accessed on 19 May 2026).

## Abbreviations

The following abbreviations are used in this manuscript:

1PC	1-point calibration
CV	Coefficient of variation
EC	Electrical conductivity
FC	Field capacity
GN	Gårdstånga Nygård
LCC	Low-cost capacitive
RMSE	Root mean square error
SO	Sensor output
SSCF	Soil specific calibration based on field data
SSCL	Soil specific calibration based on laboratory data
TH	Torna Hällestad
UCL	Universal calibration based on laboratory data
VWC	Volumetric water content
